# Structure of Mesophotic Reef Fish Assemblages in the Northwestern Hawaiian Islands

**DOI:** 10.1371/journal.pone.0157861

**Published:** 2016-07-06

**Authors:** Atsuko Fukunaga, Randall K. Kosaki, Daniel Wagner, Corinne Kane

**Affiliations:** 1 National Oceanic and Atmospheric Administration, Papahānaumokuākea Marine National Monument, Honolulu, Hawaii, United States of America; 2 Washington State University, Vancouver, Washington, United States of America; The Australian National University, AUSTRALIA

## Abstract

Mesophotic coral ecosystems (MCEs) support diverse communities of marine organisms with changes in community structure occurring along a depth gradient. In recent years, MCEs have gained attention due to their depths that provide protection from natural and anthropogenic stressors and their relative stability over evolutionary time periods, yet ecological structures of fish assemblages in MCEs remain largely un-documented. Here, we investigated composition and trophic structure of reef fish assemblages in the Northwestern Hawaiian Islands (NWHI) along a depth gradient from 1 to 67 m. The structure of reef fish assemblages as a whole showed a clear gradient from shallow to mesophotic depths. Fish assemblages at mesophotic depths had higher total densities than those in shallower waters, and were characterized by relatively high densities of planktivores and invertivores and relatively low densities of herbivores. Fishes that typified assemblages at mesophotic depths included six species that are endemic to the Hawaiian Islands. The present study showed that mesophotic reefs in the NWHI support unique assemblages of fish that are characterized by high endemism and relatively high densities of planktivores. Our findings underscore the ecological importance of these undersurveyed ecosystems and warrant further studies of MCEs.

## Introduction

Mesophotic coral ecosystems (MCEs) are characterized by the presence of light-dependent corals and associated fauna at depths below 30 m and can extend to depths of up to 150 m in locations with high water clarity [1–4]. Due to their depths, MCEs are less likely to be affected by natural and anthropogenic stressors such as storms, thermal stress and overfishing than their shallow-water counterparts [3, 5, 6]. Additionally, MCEs may be more stable over ecological and evolutionary time than shallow-water reefs [7–9]. Because of these characteristics, mesophotic habitats have received increased attention regarding their potential to serve as refugia for shallow-water (<30 m) reef organisms by serving as a source of propagules and recruits, thereby enhancing the resilience of shallow coral reefs [3, 9–11]. One of many challenges in studying MCEs is the limitation inherent to working at depths greater than those of conventional SCUBA diving (>30 m). While advances in technologies have allowed researchers to survey these habitats using mixed-gas technical diving, underwater vehicles and baited remote underwater video, faunal distributions and community structures of MCEs remain largely unknown, particularly in the Indo-Pacific [12–15].

Mesophotic habitats support a diversity of marine organisms with reported changes in community structure along depth gradients [4, 14, 16–19]. Benthic communities of MCEs mainly consist of corals, macroalgae, sponges and bryozoans [16, 20]. The amount of live benthic cover generally decreases with depth [16, 17], and the dominant taxa change from photosynthetic organisms at shallower portions to obligate heterotrophs at greater depths (reviewed in Kahng et al. [4]). Similarly, species richness of fish decreases with depth [21, 22]. Total abundance of fish generally peaks at greater depths on mesophotic reefs due to an increase in the abundance of planktivores [21], while abundances of herbivores decline sharply with depth and are relatively low in MCEs [22–24]. To date, only limited studies have focused on the ecological structure of mesophotic coral reef fishes [21–23, 25, 26], and with the exception of Parrish and Boland’s upper-mesophotic (30–40 m) study [27], none of these studies were conducted in Hawai‘i.

Spatial variability in the structure of reef fish assemblages is explained, at least in part, by environmental factors [28, 29]. These factors include depth, substrate type and complexity, water motion, and distance from shore [25, 28, 30, 31]. Physical factors correlated with changes in depth (light levels, water temperature, hydrodynamics and sedimentation) can all affect the distributions of benthic organisms in mesophotic habitats [4, 9, 14, 32]. Changes in the distributions of benthic organisms such as corals and algae may, in turn, alter the distribution of fish that rely on these organisms for shelter and food [21, 22].

**Fig 1:**
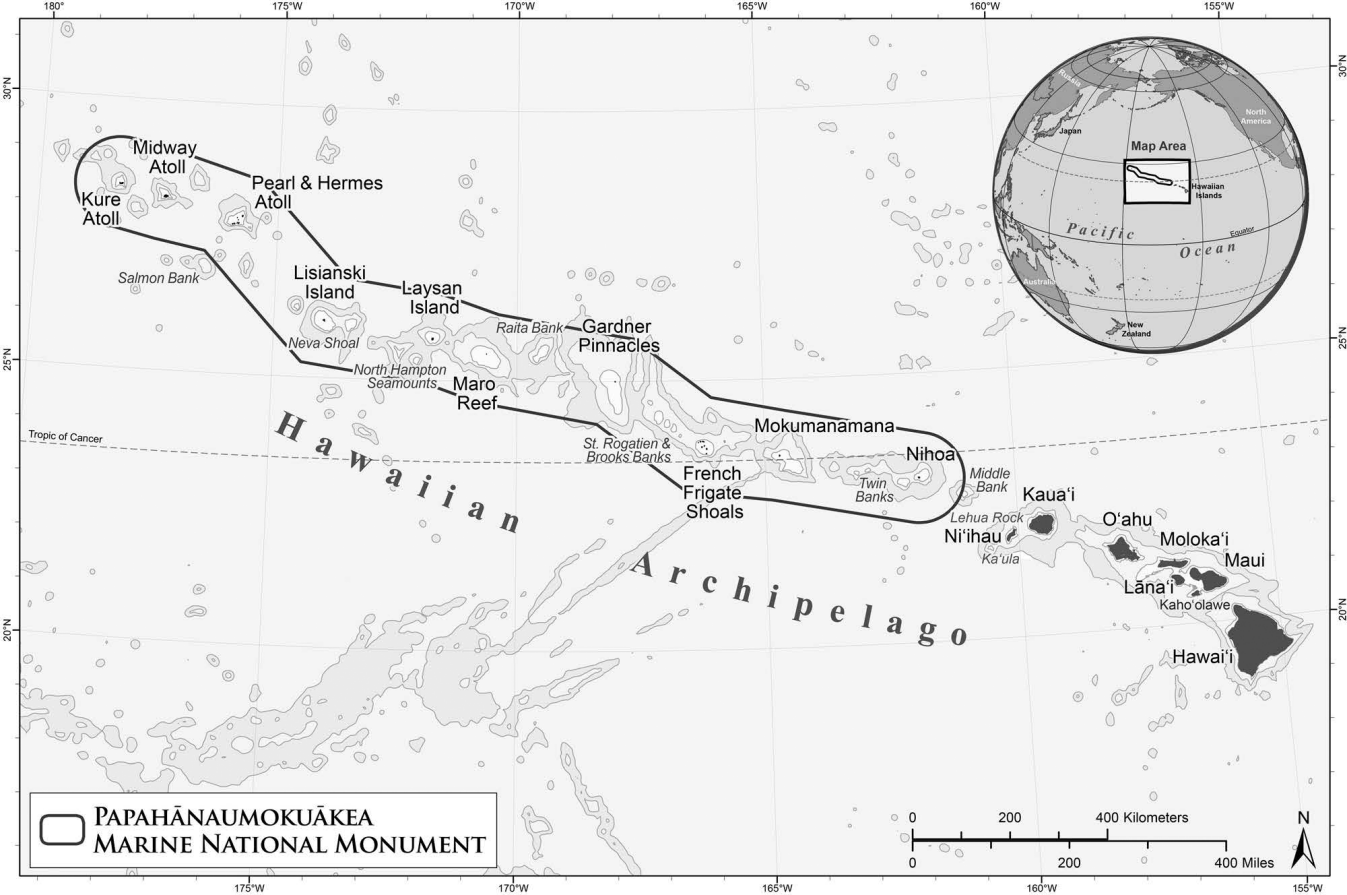
Map of the Hawaiian Archipelago including the Northwestern Hawaiian Islands.

The Northwestern Hawaiian Islands (NWHI) consist of ten major islands or atolls that span approximately 2,000 km and are located northwest of the inhabited main Hawaiian Islands (Fig 1). They are part of the Papahānaumokuākea Marine National Monument and UNESCO World Heritage Site, a marine protected area encompassing over 362,000 km^2^ that is one of the largest in the world. Previous mesophotic studies in the NWHI focused on the taxonomy, geographic ranges and sexual reproduction of black corals [18, 33–36], on the distribution of benthic communities [17], and on endemism among mesophotic reef fish assemblages [37, 38]. Here, we investigated changes in the composition and trophic structure of reef fish assemblages with depth in the NWHI. It was hypothesized that the species composition of reef fish assemblages would change with depth and, therefore, fish assemblages in MCEs would differ in structure from those in shallower waters (<30 m). It was also hypothesized that the total density of reef fish and density of planktivores would increase with depth and peak at a mesophotic depth as previously reported in other locations, while the density of herbivores would decrease with depth.

## Materials and Methods

### Ethics Statement

Field work in the Papahānaumokuākea Marine National Monument (Northwestern Hawaiian Islands) was conducted under Papahānaumokuākea Marine National Monument research permits PMNM-2010-031, PMNM-2011-042, and PMNM-2012-025.

### Survey design

All mesophotic surveys were performed using open-circuit trimix diving during three expeditions to the NWHI aboard the NOAA ship *Hi‘ialakai* in 2010 (HA-10-05, July 22 –August 20), 2011 (HA-11-05, August 29 –September 22) and 2012 (HA-12-05, September 4–28). More specifically, surveys were performed at seven islands or atolls in the NWHI including Nihoa (23°04'N, 161°55'W), French Frigate Shoals (23°52'N, 166°17'W), Gardner Pinnacles (25°01'N, 167°59'W), Maro Reef (25°25'N, 170°35'W), Lisianski (26°04'N, 173°58'W), Pearl and Hermes Atoll (27°56'N, 175°44'W) and Midway Atoll (28°12'N, 177°21'W) (Fig 1). As a part of the Papahānaumokuākea Marine National Monument, these remote islands/atolls are largely uninhabited and closed to fishing, and access is limited through a permit system. Due to this protected status, the NWHI are considered to be a relatively pristine control site for comparison to the more heavily impacted main Hawaiian Islands.

Multibeam sonar data were used to locate potential survey areas with hard-bottom slopes, ledges or other distinguishing features of fish habitats within allowable diving depths (<80 m). Survey sites were haphazardly chosen from these potential areas as randomization of site selection was extremely difficult due to both the limited area of hard-bottom habitat in appropriate depth ranges and limited availability of multibeam sonar bathymetry. Some sites were found not to be strictly hard-bottom after descending to the sites. In these cases, divers still performed fish surveys (see below) at the sites, as moving to another site was not logistically possible due to the nature of diving at mesophotic depths.

At each survey site, all conspicuous, diurnally-active fishes (minimum size of 1 cm) were counted and identified to the lowest possible taxonomic level along a 25×2 m belt transect [37, 39]. Habitat and substrate types, including the presence of algae and depth, were also recorded at each site. Surveyed mesophotic habitats were mostly hard-bottom substrates as intended, and included ledges, pavement, rubble, boulders, spur and groove, sand, *Microdictyon* spp. and other macroalgal covered slopes, as well as combinations of these habitat types. Out of 90 surveys completed in mesophotic habitats during the three expeditions, only one was performed over a completely sandy bottom. We included this survey in our data analyses as sand was one of the substrate types observed at some other sites during our surveys, and the statistical methods we used allow for robust analyses of relationships between fish density and depth in the presence of habitat variability (see Statistical analyses for details). Macroalgae were present at approximately 30% of the surveyed sites.

For comparison, we used shallow-water (1–28 m) data collected on coral reef habitats of the same seven islands by counting fish in a circular plot 15 m in diameter [40] during the NWHI Reef Assessment and Monitoring Program (RAMP) cruise to the NWHI on the NOAA ship *Hi‘ialakai* (Cruise HA-11-03, July 23 –August 21, 2011). While these shallow-water surveys used a stationary point count (SPC) method, observers swam through their circular plots at the end of their surveys in order to record small, generally site-attached species, thus reducing the possibility of missing such small-bodied fishes that could have been captured along a belt transect (non-stationary) survey. The surveys utilized bathymetric and bottom composition maps to select survey sites randomly. Similar to the mesophotic surveys, these shallow-water surveys targeted hard-bottom fish habitats, including pavement, rubble, boulders and spur and groove. Benthic compositions consisted of hard corals, macroalgae, crustose coralline algae, turf algae and sand. The highest percent cover of sand recorded at a survey site during the RAMP cruise in 2011 was 65%. Although benthic compositions change with depth [4], we consider that habitats surveyed in the mesophotic and shallow-water surveys were comparable in terms of habitat complexity and vertical relief of substratum architecture. Thus, in total, data consisted of 200 fish surveys that were categorized according to survey types (mesophotic belt transect or shallow RAMP SPC survey), depth ranges and survey years, leading to four groups: (1) deep 2010 (mesophotic, 39–67 m), (2) deep 2012 (mesophotic, 52–67 m), (3) mid-depth 2011 (mesophotic, 27–40 m) and (4) shallow 2011 (RAMP, 1–28 m) (Table 1). For all the sites surveyed, the closest sites in different depth groups were 350 m apart for the shallow and mid-depth groups and 450 m apart for the mid-depth and deep groups. The furthest sites in different depth groups within each island/atoll were 8 km apart for the shallow and mid-depth groups and 23 km apart for the mid-depth and deep groups.

**Table 1:** The number of fish surveys at each island/atoll during four separate cruises to the NWHI.

Group	Nihoa	FFS	Gardner	Maro	Lisianski	PHA	Midway	Cruise / Depth range
**Deep 2012**	2	2	2	3	4	9	1	Mesophotic / 52–67 m
**Mid-depth 2011**	-	7	7	3	9	8	-	Mesophotic / 27–40 m
**Deep 2010**	2	10	-	-	-	11	10	Mesophotic / 39–67 m
**Shallow 2011**	8	8	12	25	9	18	30	RAMP / 1–28 m

Surveyed islands/atolls were Nihoa, French Frigate Shoals (FFS), Gardner Pinnacles (Gardner), Maro Reef (Maro), Lisianski, Pearl and Hermes Atoll (PHA) and Midway Atoll (Midway).

### Statistical analyses

Analyses of reef fish assemblages were done using the software package PRIMER 6 [41] with the PERMANOVA+ add-on [42]. Due to the differences in sizes of survey areas between mesophotic and shallow-water surveys (50 m^2^ and 176.6 m^2^, respectively), data were first standardized by converting fish counts to densities (number per 100 m^2^). The structure of fish assemblages as a whole was examined using the Bray-Curtis similarity measure after square-root transformation of the density data. Differences in the structure among the four depth-year groups (deep 2012, deep 2010, mid-depth 2011and shallow 2011) were examined by analysis of similarity (ANOSIM) [43] with 4999 permutations (α = 0.05), followed by ANOSIM pairwise tests if significant differences were detected in the global test. We used similarity percentage analysis (SIMPER) [43] to determine fish species that characterized each group. The Bray-Curtis similarities were calculated among observations within each depth-year group and broken down into contribution from each species. Similarity contributions of each species were then averaged. A fish species with a high average similarity contribution (${\bar{S}}_{i}$) and a low standard deviation (*SD*(*S*_*i*_)) indicates that the species is found at a high and consistent density [44], and therefore typifies that particular depth-year group.

Changes in the structure of fish assemblages as a whole were formally analyzed along a continuous depth gradient using canonical analysis of principal coordinates (CAP) [45, 46] after pooling data from all the depth-year groups. CAP analysis finds an axis through the multivariate cloud of points that has the strongest correlation with the environmental variable of interest (i.e. depth in this case), thus allowing one to elucidate a particular relationship between the assemblage structure and the environmental variable in the presence of other potentially important variables [47]. This method was useful as our data consisted of observations from different habitat types (see Survey Design for descriptions of habitat types) collected over a relatively large geographical range. To avoid over-parameterization, the appropriate number of principal coordinate axes (*m*) was chosen for the canonical analysis by minimizing a leave-one-out residual sum of squares.

Univariate analyses of fish density and depth were done using the statistical software R (v. 3.2.2). Specifically, we analyzed the total fish density and densities by trophic categories. Fish were categorized by seven trophic habits based on various databases and references including FishBase (ver. 02/2014, www.fishbase.org), the NOAA Pacific Islands Fisheries Science Center’s database (www.pifsc.noaa.gov/cred/fish.php#), Hiatt and Strasburg [48], Hobson [49] and Hoover [50]; categories included herbivore, omnivore, corallivore, invertivore, piscivore, planktivore and apex predator. When no trophic information was available for a particular species, we used information of similar-sized congeners with similar feeding habits.

Changes in densities were modeled separately along the depth gradient using nonparametric quantile regression spline of 0.90 quantile using the function rq() in the *quantreg* package. This method can be used to model the relationship between depth and upper quantiles of fish densities and is useful when species-environment relationships show asymmetrical, non-linear and heterogeneous scatter [47]. Similar to the use of CAP analysis, this method was particularly appropriate as our data consisted of observations from different habitat types in the NWHI. The models used the function bs() in the *splines* package to construct B-spline basis expansion and to fit piecewise polynomial. The appropriate degree for the polynomial was determined based on the small-sample-size corrected version of Akaike’s information criterion (AICc). The models were then used to estimate the depths at which fish densities would reach the maximum. We obtained 90% bootstrap confidence intervals (90% CI) for each estimate by 1000 bootstrap re-sampling and re-application of the model using the polynomial degree chosen for the original data. Code for selecting and fitting models in R is provided as Supporting Information (S1 Text).

There are some important caveats resulting from the differences in survey methods between the mesophotic and shallow-water data. Previous studies on methods of fish surveys have shown that differences in methods (transect *versus* SPC) and sizes of survey areas may or may not have statistically significant and sometimes species-specific effects on identifications and counts of fishes [51, 52]. If either survey method in our study consistently counts more fish over the other, this will affect the standardization of counts to densities and thus result in a shift in quantile regression curves in either the 0–28 m (SPC method) or 27–67 m (transect method) depth range, although patterns observed within each depth range are unaffected. We do not have, however, any reasons to believe that such a systematic difference exists between the two methods, as the SPC method used in the shallow-water surveys involves swimming to record small-bodied fishes, and fishes in the NWHI do not seem to be repelled by the presence of divers, possibly due to the general absence of humans and extractive activities at these remote islands/atolls [53]. A previous study that surveyed coral reef fishes on reefs that were closed to fishing or were lightly fished also did not find statistically significant differences in standardized densities between transect and SPC surveys [52].

Another concern related to the differences in survey methods is underestimation of the number of species per site in the mesophotic surveys, relative to the shallow-water surveys, due to their smaller survey area. This will affect calculation of Bray-Curtis similarity between shallow-water and mesophotic observations, but the direction of this effect is dependent on the identity of a species missed in the mesophotic observation. If the species missed in the mesophotic observation is also present in the shallow-water observation, it results in underestimating Bray-Curtis similarity, but if it is not, Bray-Curtis similarity is overestimated. This will add variability to the data analyses when comparing shallow-water and mesophotic surveys, thus requiring some caution especially when interpreting results of ANOSIM between-group analyses (i.e. shallow *versus* mid-depth sites or shallow *versus* deep sites).

## Results

In total, 148 fish taxa were identified during 90 mesophotic surveys and 192 fish taxa during 110 shallow-water surveys (S2 Text). Of the 148 fish taxa found in mesophotic habitats, 31 taxa were unique to the mesophotic surveys. Most taxa were identified to species, but nine taxa (4%) were identified only to the family level. Significant differences in the structure of fish assemblages on the basis of the Bray-Curtis measure were detected among the four depth-year groups (deep 2012, deep 2010, mid-depth 2011 and shallow 2011; R = 0.76, p = 0.0002). Shallow 2011 and mid-depth 2011 assemblages were significantly different from one another and from either the deep 2010 or deep 2012 assemblages (Table 2). There was no statistically significant difference between the deep 2010 and deep 2012 assemblages (Table 2), and therefore we pooled these two sets for SIMPER analysis, thus categorizing the data into three depth groups: deep (mesophotic, 39–67 m), mid-depth (mesophotic, 27–40 m) and shallow (RAMP, 1–28 m).

**Table 2:** Results of ANOSIM pairwise tests.

	Mid-Depth 2011	Deep 2010	Deep 2012
**Shallow 2011**	R = 0.70, p = **0.0002**	R = 0.91, p = **0.0002**	R = 0.94, p = **0.0002**
**Mid-Depth 2011**		R = 0.33, p = **0.0002**	R = 0.30, p = **0.0002**
**Deep 2010**			R = 0.04, p = 0.096

Pairwise tests were based on the Bray-Curtis similarity calculated from square-root transformed data. Significant p-values are printed in bold (α = 0.05).

Average similarities of the structure of fish assemblages decreased from the shallow to deep groups (Table 3). At the shallow sites, the top five species of fish that typified the depth group included three herbivores (*Stegastes fasciolatus*, *Acanthurus triostegus* and *Acanthurus nigroris*) and two invertivores (*Thalassoma duperrey* and *Parupeneus multifasciatus*), while those at the deep sites consisted of four planktivores (*Pseudanthias thompsoni*, *Chromis verater*, *Chaetodon miliaris* and *Genicanthus personatus*) and one invertivore (*Parapercis schauinslandi*). At the mid-depth sites, two invertivorous species (*P*. *multifasciatus* and *T*. *duperrey*) were shared with the shallow sites, in addition to a planktivore (*Chromis hanui*), an herbivore (*Centropyge potteri*) and an invertivore (*Sufflamen bursa*). Overall, these top five species of the shallow, mid-depth and deep groups accounted for 37.7%, 48.2% and 55.7%, respectively, of the within-group similarities (Table 3).

**Table 3:** Top five fish species that typified each depth group based on SIMPER analysis on the Bray-Curtis similarity calculated from square-root transformed data.

Shallow (32.98)	Mid-depth (23.65)	Deep (15.20)
*T*. *duperrey* (5.53, 1.98, 16.8%)	*C*. *hanui* (4.04, 0.96, 17.1%)	*P*. *thompsoni* (3.39, 0.48, 22.3%)
Invertivore	Planktivore	Planktivore
*S*. *fasciolatus* (2.11, 0.85, 6.4%)	*P*. *multifasciatus* (2.24, 0.74, 9.5%)	*C*. *verater* (1.47, 0.44, 9.7%)
Herbivore	Invertivore	Planktivore
*A*. *triostegus* (1.78, 0.86, 5.4%)	*C*. *potteri* (1.98, 0.74, 8.4%)	*C*. *miliaris* (1.35, 0.47, 8.9%)
Herbivore	Herbivore	Planktivore
*A*. *nigroris* (1.52, 0.99, 4.6%)	*T*. *duperrey* (1.66, 0.59, 7.0%)	*P*. *schauinslandi* (1.32, 0.24, 8.7%)
Herbivore	Invertivore	Invertivore
*P*. *multifasciatus* (1.50, 1.31, 4.6%)	*S*. *bursa* (1.47, 0.70, 6.2%)	*G*. *personatus* (0.94, 0.39, 6.2%)
Invertivore	Invertivore	Planktivore

The heading of each column shows the depth group and the average Bray-Curtis similarity in the structure of fish assemblages in that group (within-group similarity). Each row shows the name of the species with the average similarity contribution, the ratio of the average similarity contribution to the standard deviation of similarity contribution, and the percentage of the contribution by the species to the within-group similarity in parentheses (${\bar{S}}_{i},\;{\bar{S}}_{i}/SD(S_{i})$, $\%{\bar{S}}_{i}$). Trophic categories are listed under the species names.

**Fig 2:**
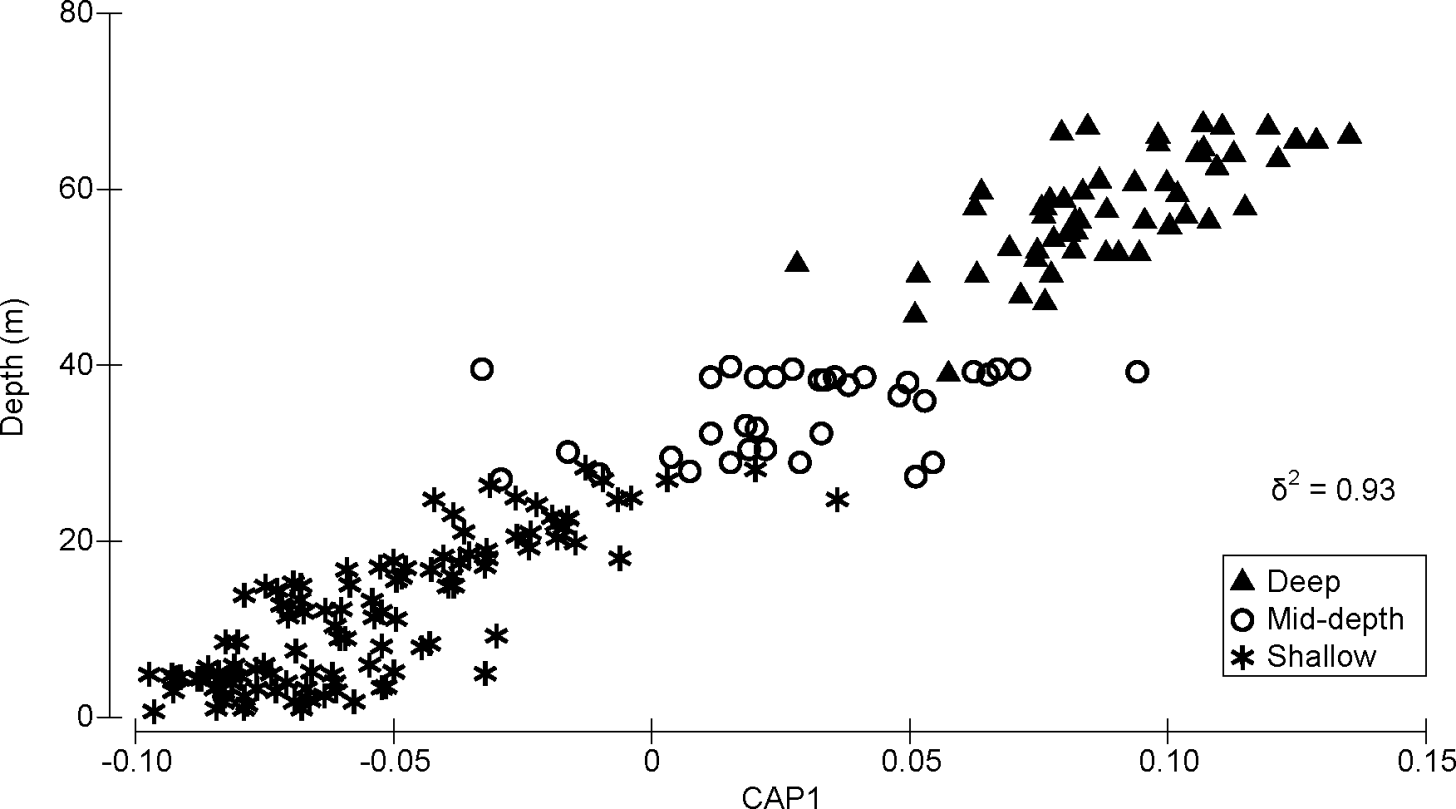
CAP ordination showing the relationship between the structure of fish assemblages and depth. Analysis was based on the Bray-Curtis measure calculated from square-root transformed fish density (100 m^-2^) using m = 33 principal coordinate axes.

There was a clear gradient from shallow to deep assemblages showing a strong relationship between the structure of fish assemblages as a whole and depth among the 200 surveys analyzed, with a canonical correlation of δ^2^ = 0.93 using *m* = 33 principal coordinate axes (Fig 2). There were, however, clear overlaps in canonical coordinate scores for observations from the mid-depth group and those from the other two groups (Fig 2).

**Fig 3:**
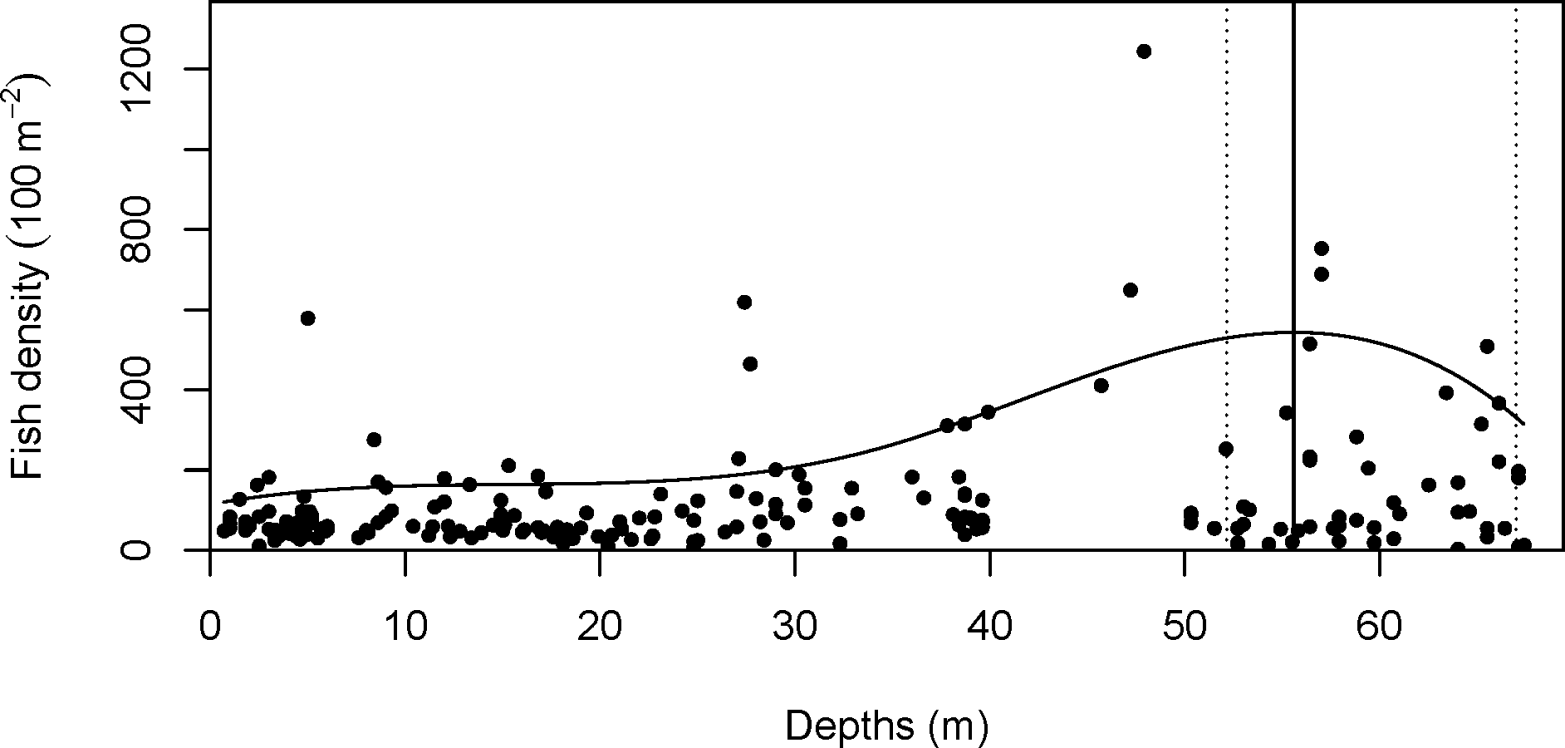
Relationship between the total fish density and depth. The regression spline model for the 90th percentile is shown, with the maximum from the model indicated by a vertical solid line and the 90% bootstrap confidence interval with dotted lines.

**Fig 4:**
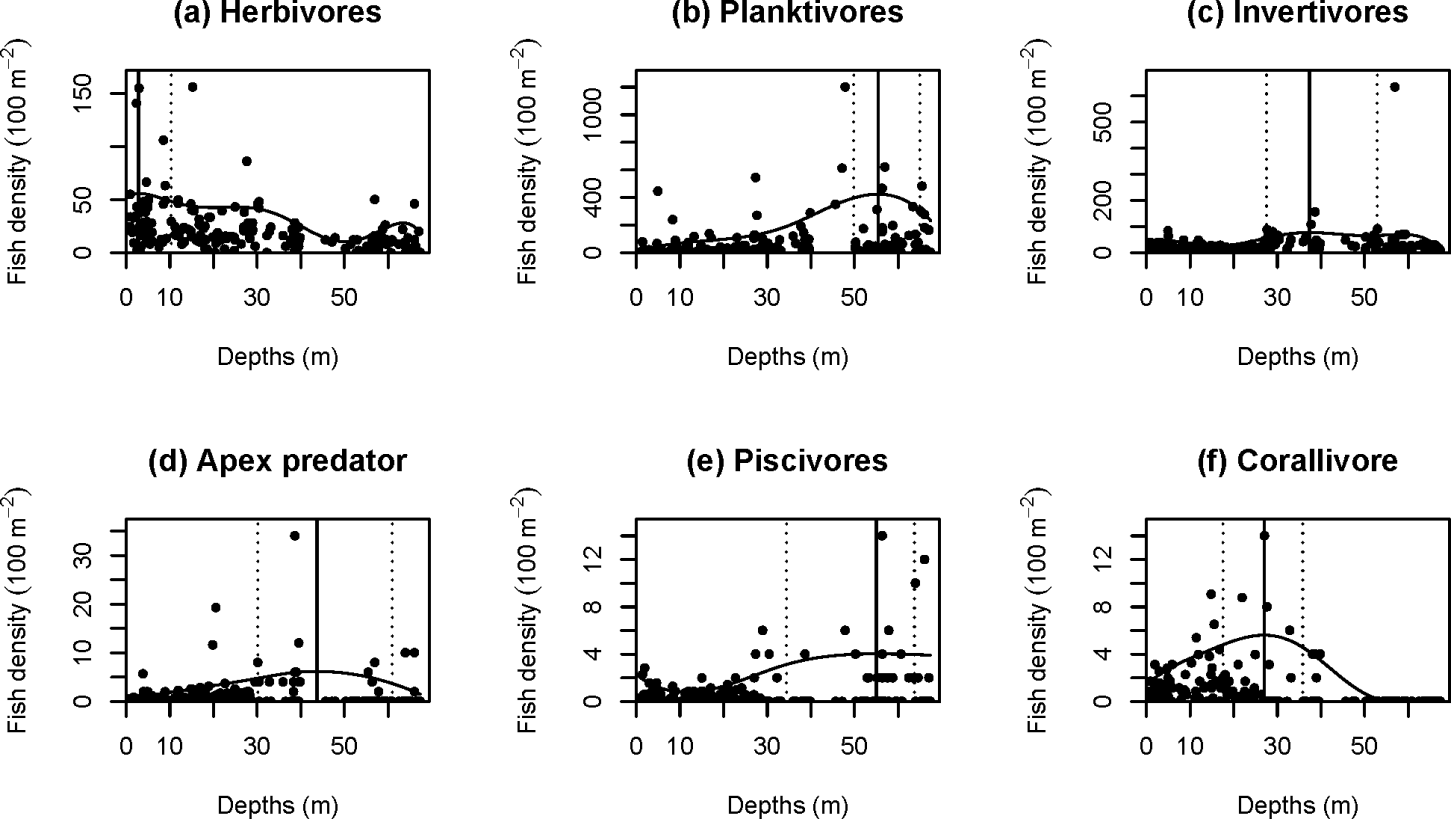
Relationship between densities of fish in each trophic category and depth. The regression spline model for the 90th percentile is shown, with the maximum from the model indicated by a vertical solid line and the 90% bootstrap confidence interval with dotted lines.

Total fish density increased at the mid-depth and deep sites and reached a maximum at 55.6 m (90% CI = 50.9–67.3 m; Fig 3). Distributions of fish by trophic categories were consistent with the trophic categories of the fish that typified the three depth groups (Fig 4). Herbivores reached a maximum density at 2.8 m (90% CI = 0–12.2 m) and gradually decreased with depth (Fig 4A), while densities of planktivores reached a maximum at 55.4 m (90% CI = 50.1–65.0 m; Fig 4B). Densities of invertivores were slightly higher at the mid-depth and deep sites than at the shallow sites, reaching a maximum at 37.4 m (90% CI = 27.5–53.1 m; Fig 4C). Apex predators reached their maximum density at 43.7 m (90% CI = 30.7–62.1 m; Fig 4D), piscivores at 55.0 m (90% CI = 35.0–63.5 m; Fig 4E) and corallivores at 27.0 m (90% CI = 18.5–35.5 m; Fig 4F), but overall, these latter three trophic groups were not very abundant even at their maximum densities. Omnivores were rare at all depths, with an overall mean density of only 0.20 per 100 m^2^.

## Discussion

Mesophotic fish assemblages in the NWHI were characterized by higher densities of planktivores and invertivores, as well as by lower densities of herbivores when compared to assemblages on shallow reefs (<30 m). In shallow habitats, herbivores and invertivores were equally abundant. These findings are consistent with the results of a previous study in the main Hawaiian island of Kauaʻi by Friedlander and Parrish [29]. Although the depth range of that study was limited to <13.6 m, herbivores were abundant in shallow reef habitats and planktivores along deep reef slopes. Low densities of herbivores at mesophotic depths are also consistent with previous studies in the Marshall Islands [21] and the Red Sea [24]. Furthermore, high abundances of planktivores have also been reported during mesophotic studies in the Trindade and Martim Vaz island group [54], the Marshall Islands [21] and the Wai‘anae coast of O‘ahu, Hawai‘i [55]. The high density of planktivores in the present study contributed to the higher overall density of fish at mesophotic depths, as total fish density and the density of planktivores both reached their maximum at 55 m. These two quantile regression curves (planktivores and total fish density) had very similar shapes at mesophotic depths, with an increase starting around 30 m, reaching the maximum at 55 m and decreasing to 67 m (Figs 3 and 4B).

In the present study, macroalgae were observed at approximately 30% of the mesophotic sites surveyed (see Survey Design for descriptions of the surveyed habitats). While decreases in light levels in mesophotic habitats are expected to limit distributions and growth rates of algae [32, 56], benthic macroalgal cover in the NWHI has previously been reported to be the highest in the 40–50 m depth range and above 20–30% at 70 m [17]. Relatively high abundances of macroalgae at mesophotic depths are not unique to the NWHI, but have also been reported in nearly every mesophotic environment surveyed [5, 24, 54]. The effects of depth on distributions of fish occurring through modifications of benthic communities that serve as shelter and/or food for fish has previously been discussed by Pereira-Filho et al. [54]. The scarcity of herbivores in the presence of macroalgae observed during this study on mesophotic reefs suggests that the distribution of herbivores is regulated by factors other than food availability. Temperature has been proposed as an environmental factor that may restrict the geographic ranges of fishes along latitudinal gradients due to physiological processing constraints on species with diets high in plant material [57, 58]. A similar mechanism may restrict herbivorous fishes to warmer end of the temperature gradient between shallow and deep coral reefs.

Despite the overlaps between fish species found at the mid-depth sites and those found at both the shallow and deep sites, different species were dominant at the shallow and deep sites (Table 3), suggesting that individual species are adapted to specific environmental conditions. While the ultimate drivers of the differences in the structure of fish assemblages observed during this study remain unknown, temperature may explain, at least in part, why each depth group was composed of a characteristic group of species. Temperature is considered to be one of the primary factors limiting the distributions of ectothermic organisms with depth [59–61]. The top of the thermocline shoals to shallower depths as one moves northward along the Hawaiian Archipelago, and it is found at depths shallower than 60 m at the northern atolls of Midway and Pearl and Hermes [62]. Approximately 45% of the mesophotic surveys of this study were performed at those two atolls (Table 1), and therefore deep-zone fish assemblages of those atolls are likely exposed to lower temperatures, for longer periods of time, than similar deep assemblages on lower-latitude Hawaiian reefs. Average water temperature has been reported to range from 21 to 24°C at 50–65 m depths at these two northern atolls, whereas temperatures as warm as 27°C have been recorded at similar depths off Maui and Oahu (20°N and 21°N, respectively) [62].

High levels of endemism among mesophotic fish assemblages in the NWHI have previously been reported [37, 38]. In the present study, fishes that typified the mid-depth and deep groups included species endemic to the Hawaiian Islands [50]: four planktivores (*Chaetodon miliaris*, *Chromis hanui*, *Chromis verater* and *Genicanthus personatus*), one invertivore (*Thalassoma duperrey*) and one herbivore (*Centropyge potteri*) (Table 3). These endemic species contributed to 32.5% and 24.7% of the within-group similarities at the mid-depth and deep sites, respectively. *C*. *potteri* and *T*. *duperrey* have previously been reported to be highly abundant in outer drop-off habitats that extended from the depth of approximately 25 m to much greater depths on the island of Hawaiʻi [49]. In the same study, *Pseudanthias thompsoni*, *C*. *verater* and *C*. *miliaris*, all diurnal planktivores, were also recorded, though generally in lower abundances. Using submersibles, Brock and Chamberlain [55] found that *C*. *verater* and *C*. *militaris* were the dominant species on deep-water escarpments off the island of Oʻahu. In addition, the lower average similarity in the structure of fish assemblages in the deep group compared to that of either the shallow or mid-depth group indicates that fish assemblages at the deep sites were more variable. Accordingly, beta diversity measured by multivariate dispersion [63] (which is, in our case, the average distance of individual observations to their depth group centroid defined in the space of Bray-Curtis similarity) increased from the shallow group (47.3 ± 0.8; mean ± standard error) to mid-depth (53.4 ± 1.1) and deep (59.8 ± 1.0) groups.

Given the dominance of herbivores and planktivores on shallow and deep reefs respectively, it is possible that shallow and deep coral reefs in the NWHI are supported by substantially different sources of primary productivity. Stable isotope analyses can shed light on energy pathways through coral reef ecosystems [64, 65] and have shown that shallow reef fish assemblages in the NWHI exhibit a strong signal of benthic primary productivity [66]. This is consistent with our study’s finding of numerical dominance by herbivorous species in shallow water. Future stable isotope studies may find that planktivore-dominated mesophotic fish assemblages are supported by planktonic rather than benthic sources of primary productivity. These studies of nutrient cycling will increase our understanding of ecological linkages among ecosystem components of the NWHI and help us develop effective management strategies to enhance protection of both shallow and deep reef ecosystems of the Papahānaumokuākea Marine National Monument.

The concept of mesophotic reefs as refugia for shallow reef ecosystems has dominated the literature in recent years with the premise that mesophotic reefs may serve as refuge areas and potential sources of propagules for shallow reefs [9–11]. While the idea of deep reefs sourcing propagules to replenish degraded shallow systems has garnered widespread interest, explicit tests to date have been conducted mostly on coral species and with mixed results [67–72]. The changes in the assemblage and trophic structures of fish observed in our study support the refugia hypothesis only to a limited extent; upper mesophotic reef fish assemblages (i.e. assemblages at the mid-depth sites) have some overlaps in species composition with shallow reefs. At depths below this, shallow and mesophotic fishes are largely distinct from one another, and thus the potential for replenishment of shallow reefs from mesophotic depths is limited. Even at upper mesophotic depths, refuge potential is limited mostly to invertivores and planktivores. Therefore, replenishment from mesophotic depths may aid in recovery of fish densities, but not functional assemblages to completely restore degraded shallow reef systems.

Variations in the structure of fish assemblages are explained by multiple environmental factors [29]. The present study demonstrated clear relationships between depth and the structure of fish assemblages in the NWHI. The uses of quantile regression and CAP analysis to model these relationships were helpful since other unmeasured factors could affect distributions of the organisms [47, 73]. Quantile regression showed changes in the maximum attainable density of each trophic category along a depth gradient in the presence of other factors potentially affecting fish distribution. CAP analysis demonstrated gradual changes in the structure of fish assemblages as a whole with depth; as suggested by the overlaps in canonical coordinate scores for observations from the mid-depth zone and those from the other two zones, the mid-depth zone in the present study seems to be a transition zone. Consistent with this observation, several previous studies suggest that the arbitrary upper depth limit attributed to MCEs (i.e. 30 m) does not represent a static physiological or ecological boundary for marine organisms, and simply corresponds to the limit of conventional SCUBA diving (reviewed by Kahng et al. [4]).

As previously mentioned, a comparison of fish assemblages between shallow and mesophotic habitats in the present study requires some caution due to the differences in survey methods. However, the CAP analysis showed a clear overlay of the assemblage structures from both survey methods around 27–28 m depths where the depth ranges of the shallow (RAMP 2011 cruise) and mid-depth (Mesophotic 2011 cruise) sites overlapped. Furthermore, *post hoc* comparison of these observations (five sites in the shallow group at 26.4–28.4 m depths and four sites in the mid-depth group at 27.1–28.0 m depths) using ANOSIM [43] showed no statistically significant differences between the survey methods (*R* = 0.3, p = 0.15) in terms of the assemblage structure on the basis of Bray-Curtis similarity using square-root transformed density data. This indicates that species identification and their square-root transformed densities were similar between the two methods in this depth range, as Bray-Curtis similarity accounts for both identity and abundance of each species. While we acknowledge that the overlap of the depth ranges is narrow and the number of samples compared was relatively small, this overlap of the assemblage structure, as well as consistent results between quantile regressions and SIMPER analysis (in which within-group analyses would not be affected by the presence/absence of organisms in other groups), give us some assurance that the patterns observed in the present study were not an artifact caused by differences in survey methods.

In conclusion, the value of the mesophotic habitat in the NWHI is driven by its own intrinsic attributes: high total density of fish, a distinct source of diversity that complements the diversity of shallow coral reefs and globally significant levels of endemism. As we collect more ecological data from mesophotic habitats in the NWHI, comparison of fish assemblages among different islands/atolls or different habitat types, as well as species-specific analyses, will become possible. These future studies will undoubtedly reveal new features about MCEs, and thereby increase our understanding of these important yet vastly undersurveyed ecosystems.

## Supporting Information

S1 Text R analysis code for quantile regression. (TXT)

S2 Text Density of fish per 100 m^2^ at each survey site. (TXT)
